# Impact of Benzodiazepines and Illness Duration on Obsessive–Compulsive Disorder during COVID-19 in Italy: Exploring Symptoms’ Evolutionary Benefits

**DOI:** 10.3390/brainsci14040338

**Published:** 2024-03-30

**Authors:** Giordano D’Urso, Alfonso Magliacano, Marco Manzo, Mattia Vittorio Pomes, Carla Iuliano, Felice Iasevoli, Bernardo Dell’Osso, Andrea de Bartolomeis

**Affiliations:** 1 Section of Psychiatry, Department of Neuroscience, Reproductive, and Odontostomatological Sciences, University of Naples Federico II, 80131 Naples, Italy; marcomanzo95@gmail.com (M.M.); mavipo93@gmail.com (M.V.P.); felice.iasevoli@unina.it (F.I.); adebarto@unina.it (A.d.B.); 2 IRCCS Fondazione Don Carlo Gnocchi ONLUS, 50143 Florence, Italy; a.magliacano@live.it; 3 School of Cognitive Psychotherapy (SPC), 80100 Naples, Italy; iulianocarla@gmail.com; 4 Department of Biomedical and Clinical Sciences, University of Milan, 20157 Milan, Italy; bernardo.dellosso@unimi.it; 5 Department of Psychiatry and Behavioral Sciences, Bipolar Disorders Clinic, Stanford University, Stanford, CA 94305, USA; 6 “Aldo Ravelli” Center for Nanotechnology and Neurostimulation, University of Milan, 20122 Milan, Italy

**Keywords:** obsessive–compulsive disorder, COVID-19, biological evolution, benzodiazepines, mental health, anxiety disorders, psychopathology

## Abstract

Obsessive–compulsive disorder (OCD) is believed to follow a waxing and waning course, often according to environmental stressors. During the COVID-19 pandemic, pre-existing OCD symptoms were reported to increase and to change from checking to washing behaviors, while new-onset symptoms were predominantly of the hoarding type. In the present study, we followed the evolution of OCD symptoms, anxiety, depression, and insights of illness in forty-six OCD patients throughout the pandemic. Clinical measures were collected at four different time points before and during the COVID-19 pandemic in Italy. Within-subject comparisons were used to compare clinical scale scores across time, and correlations were examined between patients’ baseline characteristics and changes in clinical scores. We found that all clinical measures increased during the first Italian lockdown with respect to the pre-pandemic values. Anxiety decreased during the temporary elimination of restriction provisions, whereas the severity of OCD symptoms and insight returned to pre-pandemic values during the second mandatory lockdown. These results were observed only in two sub-groups of patients: those taking benzodiazepines and those with shorter illness duration. Our findings suggest the need for additional clinical attention to these specific sub-groups of OCD patients in case of particularly distressing circumstances while pointing to a possible adaptive role of their OCD symptoms when the environment requires a higher care of hygiene and an extraordinary supply of essential resources.

## 1. Introduction

Ritualistic behaviors can occur in various forms, both in humans and in other animal species, and can range from culturally accepted and sustained behaviors to truly pathological ones, such as compulsions in humans and adjunctive behaviors in animals [1,2]. They seem to have in common the aim to cope with the unpredictability of the environmental circumstances and, at least in humans, the subjective feeling of being capable of (or responsible for) preventing a bad outcome (i.e., so-called magical thinking). In humans and in several other animal species, pathological ritualistic behaviors include motor sequences related to hygiene care and resource transport and storage. These motor sequences have been highly preserved during evolution due to their function in thermoregulation, their protective value against parasites and diseases (hygiene care), and their utility in case of shortage of essentials (resources transport and storage) [3]. In humans, these evolutionarily preserved behaviors are excessively and inappropriately exhibited by patients suffering from obsessive–compulsive disorder (OCD).

OCD is a chronic psychiatric disorder characterized by obsessions and/or compulsions. Obsessions are unwanted and intrusive thoughts, impulses, images, or urges that are persistent and repetitive, often accompanied by anxiety. Compulsions are repetitive mental acts or behaviors, typically made in response to obsessions, following rigid rules, or to achieve a sense of “completeness” [4]. The symptoms of OCD can be categorized into a five-factor model, as proposed by Stein et al. [5]. These factors include “contamination” (involving contamination or cleanliness obsessions and cleaning compulsions), “harmful thoughts” (encompassing thoughts of harm to self and others and checking compulsions), “forbidden thoughts” (including aggressive, sexual, or religious obsessions with mental rituals or praying), “symmetry" (comprising symmetry obsessions, repeating, ordering, and counting compulsions), and “hoarding” (involving hoarding or saving obsessions and related compulsions). Nationally representative surveys have confirmed that OCD has a lifetime prevalence of 2–3%, although figures vary across regions [6]. The “insight”, described as the awareness of the irrationality and/or excessiveness of obsessions and compulsions, is an important aspect of the psychopathology of OCD. In the Diagnostic and Statistical Manual of Mental Disorders (DSM-5), insight is categorized into three specifiers: good or fair insight, poor insight, and absent insight or delusional beliefs [4]. The inclusion of insight as a specifier in the DSM-5 is warranted by its clinical relevance both for severity assessment and treatment implications [7].

The COVID-19 pandemic has been linked to a substantial increase in various psychiatric disorders within the general population [8,9]. According to a recent systematic review and meta-analysis, the pandemic has caused heightened levels of anxiety, depression, psychological distress, and alcohol use disorders. The factors contributing to the global surge in the prevalence of psychiatric symptoms encompass a range of stressors associated with the pandemic. Perceived risk of infection, experiencing COVID-19-like symptoms, scarcity of masks, and ambiguous guidelines on mask use have been correlated with heightened levels of anxiety and depression. Moreover, the implementation of lockdowns and the requirement for home confinement have exacerbated mental health conditions, particularly among individuals experiencing marital or family conflicts, instances of physical or psychological abuse, and inadequate housing conditions to cope with confinement measures. Furthermore, the impact of social media on mental health during major population events has become increasingly prominent. Heavy consumption of COVID-19-related content on social media platforms has been associated with elevated levels of anxiety, depression, and acute stress. This phenomenon may be attributed to the propagation of emotional contagion, dissemination of conflicting COVID-19 information, and the proliferation of fear-inducing misinformation through online social networks [10]. In addition, the COVID-19 pandemic has disrupted mental health care delivery, necessitating adaptations in service provision. Some psychiatric units have been resized or repurposed to accommodate COVID-19 patients, while outpatient and residential activities have been partially affected, with patients resorting to alternative ways of communication, such as phone appointments and e-communications [11].

Several studies have examined the impact of the COVID-19 pandemic on OCD symptoms, and the results have been varied. Some studies have reported a worsening of symptoms, while others have found no change or even an improvement [12,13,14]. The studies reporting a worsening in OCD symptoms during the COVID-19 lockdown found an increase in obsessions related to contamination, health, safety, and compulsions of cleaning, washing, and checking [15]. This increase may be due to several factors, including increased anxiety and social isolation, as well as the new daily routines and restrictions imposed by the lockdown. Overall, 78% of studies reported an increase in OCD symptoms during the lockdown, while 22% reported no change [16]. One possible explanation for this discrepancy is the cultural context in which the different studies took place, as cultural beliefs and societal norms can significantly influence the expression and interpretation of OCD symptoms. For example, a decreased risk of OCD worsening was associated with being from Europe in Van Ameringen’s study [14], while a lack of adequate access to technology for e-visits might have affected the psychiatric care in certain geographical areas, causing a higher burden of symptoms worsening.

In Italy, two major COVID-19 epidemiological waves occurred in 2020, one in spring and another in autumn, which were accompanied by stringent restriction measures. During the inter-wave period, particularly in the summer months, there was a notable decline in infection rates, prompting the relaxation of restrictions and the resumption of normal activities. Despite the second wave being less severe than the initial one, it was likely exacerbated by heightened social interactions during the summer vacation period [17].

In a previous study from our group, we observed that the mandatory lockdown modified the characteristics of OCD symptoms, highlighting the diverse and dynamic nature of the disorder, whose psychopathological expressivity is influenced by environmental circumstances. In particular, we found a partial transition from checking to washing symptoms in those patients who had already been diagnosed with OCD before the pandemic, whilst all the patients whose symptoms onset occurred during the pandemic predominantly displayed hoarding symptoms [16].

Starting from this evidence, we posed the question of whether OCD symptoms could paradoxically become an evolutionary advantage under the deadly circumstances of the COVID-19 pandemic. In fact, although psychopathological symptoms are maladaptive by definition and impair the evolutionary fitness of the affected individuals, the COVID-19 pandemic can be considered a natural experiment in this respect, suggesting the possibility that specific OCD symptoms (i.e., hygiene-related obsessions and compulsions, hoarding behaviors) might paradoxically become an advantage under the extreme evolutionary pressure represented by a global pandemic, in which increased attention to hygiene and proneness to hoard essential goods can make the difference between life and death. Therefore, in the present study, we followed the course of symptoms of a sample of OCD patients throughout the pandemic, with the hypothesis that this could add knowledge about this potential ossimoronic, adaptive nature of OCD symptoms.

Our main aim was to investigate the possible changes in psychopathological symptoms of OCD patients throughout the different phases of the COVID-19 pandemic in the Italian cultural context. To this aim, we assessed the severity of their OCD, anxiety, and depression symptoms as well as the level of insight at four time points. Secondarily, we aimed to identify the clinical and demographic factors influencing the symptom severity changes. Among these factors, we included benzodiazepines use, in light of the evidence of increased anxiety levels in OCD patients during the COVID-19 lockdown and of a possible role of the anxious diathesis in shaping their reaction to the pandemic [18].

## 2. Materials and Methods

### 2.1. Study Design

This study is a longitudinal mixed-methods investigation conducted in Italy, aimed at elucidating the impact of the COVID-19 pandemic on the psychopathological symptoms of OCD patients.

### 2.2. Participants

During the first mandatory lockdown in Italy, our research team selected and contacted by phone 102 OCD patients, diagnosed according to DSM-5 criteria from the clinical records of the OCD outpatient clinic of the University Hospital “Federico II” in Naples, Italy. Among these, 46 (45%) agreed to participate in the study, while the remaining provided no consent due to personal reasons or were not reachable before the end of the lockdown. Inclusion criteria were: (i) patients diagnosed with OCD according to DSM-5 criteria; (ii) ages ranging from 18 to 70 years; (iii) patients who had been visited within the three months preceding the pandemic outbreak; (iv) patients found clinically stable at the time of the visit preceding the pandemic outbreak; and (v) patients providing informed consent to participate in the study. The exclusion criteria included: (i) the presence of psychiatric disorders other than OCD; and (ii) the presence of medical conditions potentially affecting the reliability of test results. The data were collected at four time points: within three months before the pandemic outbreak, i.e., from 9 December 2019 to 8 March 2020 (T0); from the ethical approval of the study to the end of the first Italian mandatory lockdown, i.e., from 22 April to 18 May 2020 (T1); during a temporary elimination of restriction provisions, i.e., from 1 June to 30 September 2020 (T2); and during the second Italian mandatory lockdown, i.e., from 1 November to 31 December 2020 (T3).

### 2.3. Measures and Procedure

Four rating scales were administered at the four time points: the Yale–Brown Obsessive–Compulsive Scale (Y-BOCS) [19], the Brown Assessment of Belief Scale (BABS) [20], the Beck Depression Inventory-II (BDI-II) [21], and the State-Trait Anxiety Inventory-Y (STAI-Y) [22].

The Y-BOCS is a standardized assessment tool widely used to measure the severity of OCD. It consists of a structured interview and a symptom checklist designed to evaluate the presence and intensity of obsessions and compulsions. The scale has demonstrated strong validity and reliability in assessing OCD symptoms, with each item rated on a scale from 0 to 4, where higher scores indicate greater severity of OCD symptoms.

The BABS is a clinician-administered scale developed to assess the strength of beliefs associated with OCD symptoms. Comprising 7 items, it targets specific beliefs related to obsessive thoughts and compulsive behaviors. The scale has been validated as a reliable measure of belief conviction in individuals with OCD. Each item is scored based on a clinician-administered assessment, with scores ranging from 0 to 4, where higher scores reflect stronger belief convictions associated with OCD symptoms.

The BDI-II is a self-reported inventory comprising 21 multiple-choice items designed to evaluate affective, cognitive, and physical symptoms associated with depression. Each item is scored on a scale from 0 to 3, reflecting the increasing severity of symptoms. The inventory has demonstrated strong validity and reliability in assessing depression symptoms.

Similarly, the STAI-Y is a widely employed instrument for measuring both trait and state anxiety. It consists of 20 items dedicated to assessing trait anxiety and another 20 items focused on evaluating state anxiety. For our study, we only assessed state anxiety. The STAI-Y has been extensively validated and exhibits high reliability in assessing anxiety symptoms.

All scales were administered in the Italian language in which they had been previously validated.

### 2.4. Statistical Analyses

The scores of the clinical scales (Y-BOCS, BABS, BDI-II, and STAI-Y) were compared across time by means of the Wilcoxon signed-rank test. The Spearman’s correlation coefficients were computed between patients’ baseline characteristics (age, sex, education, and illness duration) and the difference in the score of the clinical scales across time points. In the case of significant correlations, the OCD sample was split into two sub-samples based on the median score of the variable (greater or equal than the median value vs. smaller than the median value). We also controlled for a possible moderating effect of benzodiazepines by comparing the scores of the clinical scales in patients taking vs. not taking these drugs at T0.

A post hoc power analysis for Wilcoxon signed-rank tests was performed to calculate the achieved power using G*Power v. 3.1.9.6. The level of significance was set at 0.05. All analyses were performed by IBM SPSS v.25 (IBM Corp., Armonk, NY, USA).

## 3. Results

### 3.1. Overall OCD Group

We included an overall sample of 46 OCD patients whose characteristics are reported in Table 1 and elsewhere [18].

Patients were in treatment with combinations of benzodiazepines (*n* = 36; 78%), SRIs (*n* = 43; 93%), antidepressants other than SRIs (*n* = 3; 7%), antipsychotics (*n* = 10; 22%), antiepileptics (*n* = 7; 15%), and lithium (*n* = 3; 7%).

At the group level, we found that Y-BOCS scores increased at T1 compared to T0, although this difference only approached statistical significance (Z = −1.95; *p* = 0.05). The Y-BOCS scores did not differ between T1 and T2 (*p* > 0.05) but decreased significantly at T3 (Z = −2.412; *p* = 0.016; power = 74%).

The BABS scores increased at T1 with respect to T0 (Z = −2.199; *p* = 0.028; power = 67%), remained stable at T2, and decreased significantly at T3 (Z = −2.409; *p* = 0.016; power = 76%).

**Figure 1:**
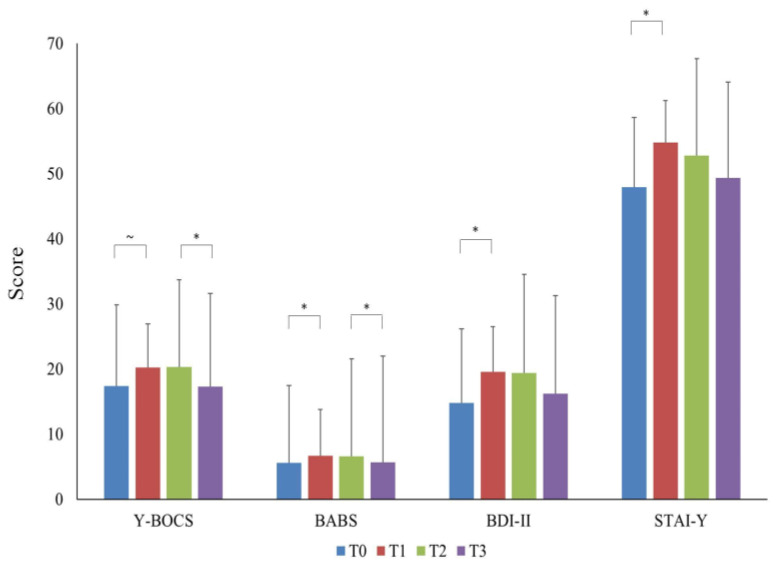
The graphs display mean Y-BOCS, BABS, BDI-II, and STAI-Y values at the four study time points in the overall OCD sample. Error bars indicate standard deviation. “*” indicates significant differences across time points. “~” indicates a quasi-significant difference (*p* = 0.05). Abbreviations: Y-BOCS = Yale–Brown Obsessive–Compulsive Scale; BABS = Brown Assessment of Beliefs Scale; BDI-II = Beck Depression Inventory-II; STAI-Y = State-Trait Anxiety Inventory-Y; T0 = three months before the pandemic outbreak; T1 = first mandatory Italian lockdown; T2 = temporary interruption of restriction provisions; T3 = second mandatory Italian lockdown.

Both the BDI-II (Z = −2.968; *p* = 0.003; power = 89%) and the STAI-Y (Z = −3.232; *p* = 0.001; power = 93%) increased significantly at T1 compared to T0, then remained stable up to T3 (all *p* > 0.05; see Figure 1).

### 3.2. Results as a Function of Benzodiazepine Use

**Figure 2:**
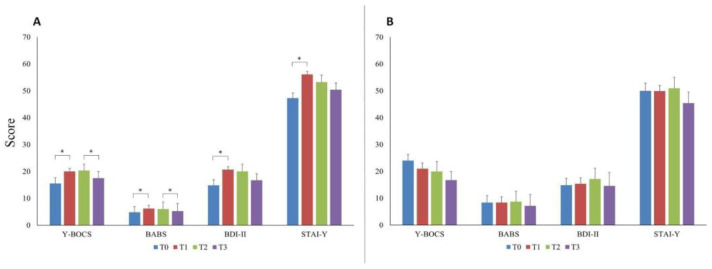
The graphs display mean Y-BOCS, BABS, BDI-II, and STAI-Y values at the four study time points in the sub-group of patients taking (**panel A**) and not taking (**panel B**) benzodiazepines. Error bars indicate standard deviation. “*” indicates significant differences across time points. Abbreviations: Y-BOCS = Yale–Brown Obsessive–Compulsive Scale; BABS = Brown Assessment of Beliefs Scale; BDI-II = Beck Depression Inventory-II; STAI-Y = State-Trait Anxiety Inventory-Y; T0 = three months before the pandemic outbreak; T1 = first mandatory Italian lockdown; T2 = temporary interruption of restriction provisions; T3 = second mandatory Italian lockdown.

In patients taking benzodiazepines (*n* = 36), the Y-BOCS, the BABS, the BDI-II, and the STAI-Y scores showed the same trend that we observed in the overall OCD group. In particular, the Y-BOCS (Z = −2.581; *p* = 0.010; power = 80%) and the BABS (Z = −2.199; *p* = 0.028; power = 68%) increased significantly at T1, remained stable at T2, and then decreased at T3 (Z = −2.202; *p* = 0.028; power = 68% and Z = −2.108; *p* = 0.035; power = 64%, respectively). Similarly, the BDI-II and the STAI-Y increased significantly at T1 (Z = −2.865; *p* = 0.004; power = 87% and Z = −3.559; *p* < 0.001; power = 96% respectively; Figure 2A). Conversely, no significant differences in any scale and any time point were observed in patients who did not use benzodiazepines (*n* = 10; all *p* > 0.05; Figure 2B).

### 3.3. Results as a Function of Illness Duration

The illness duration correlated significantly with both the difference in the Y-BOCS scores between T0 and T1 (ρ = −0.403; *p* = 0.005) and between T2 and T3 (ρ = 0.336; *p* = 0.022), and the difference in the BABS scores between T0 and T1 (ρ = −0.349; *p* = 0.018) and between T2 and T3 (ρ = 0.439; *p* = 0.002). Similarly, illness duration correlated significantly with both the difference in the STAI-Y scores between T0 and T1 (ρ = −0.319; *p* = 0.031), whereas the correlation with the difference in the BDI-II scores between T0 and T1 and only approached statistical significance (ρ = −0.289; *p* = 0.051). Moreover, age correlated with the difference in the BABS scores between T3 and T2 (ρ = 0.375; *p* = 0.010). No further significant correlation has been observed (all *p* > 0.05).

**Figure 3:**
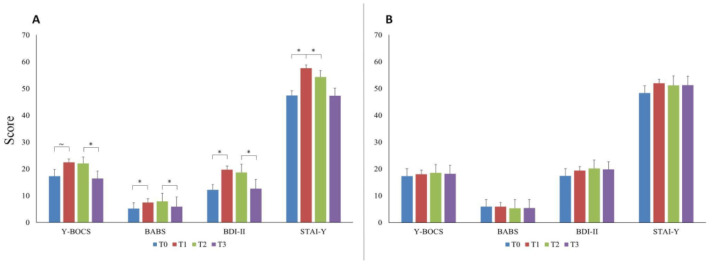
The graphs display mean Y-BOCS, BABS, BDI-II, and STAI-Y values at the four study time points in the sub-group of patients with short (**panel A**) and long (**panel B**) illness duration. Error bars indicate standard deviation. “*” indicates significant differences between time points. “~” indicates a quasi-significant change (*p* = 0.052). Abbreviations: Y-BOCS = Yale–Brown Obsessive–Compulsive Scale; BABS = Brown Assessment of Beliefs Scale; BDI-II = Beck Depression Inventory-II; STAI-Y = State-Trait Anxiety Inventory-Y; T0 = three months before the pandemic outbreak; T1 = first mandatory Italian lockdown; T2 = temporary interruption of restriction provisions; T3 = second mandatory Italian lockdown.

Once the overall OCD sample was split into two sub-samples based on the median value of illness duration, we found that in patients with a shorter illness duration (*n* = 23), the Y-BOCS and the BABS scores showed the same trend that we observed in the overall OCD group. Particularly, the BABS increased significantly at T1 (Z = −2.197; *p* = 0.028; power = 67%), whereas the Y-BOCS only showed a trend to increase (Z = −1.943; *p* = 0.052). Both Y-BOCS and BABS remained stable at T2 and decreased at T3 (Z = −2.280; *p* = 0.023; power = 70% and Z = −2.554; *p* = 0.011; power = 77%, respectively; Figure 3A).

The BDI-II and the STAI-Y, instead, showed a slightly different trend to the overall OCD group. Specifically, both increased significantly at T1 (Z = −2.779; *p* = 0.005; power = 84% and Z = −2.951; *p* = 0.003; power = 87%, respectively); thereafter, the STAI-Y decreased significantly at T2 (Z = −2.155; *p* = 0.031; power = 65%) and remained stable at T3 (*p* = 0.211), whereas the BDI-II only decreased at T3 (Z = −2.239; *p* = 0.025; power = 69%; Figure 3A). No significant differences in any scale and any time point were observed in patients with longer illness duration (*n* = 23; all *p* > 0.05; Figure 3B). Importantly, out of the 36 patients treated with benzodiazepines, only 15 (42%) had a shorter illness duration; out of the 23 patients with shorter illness duration, 15 (65%) were treated with benzodiazepines, and 8 (35%) were not; out of the 23 OCD patients with longer illness duration, 21 (91%) were treated with benzodiazepines, and 2 (9%) were not.

## 4. Discussion

The main results of the present study are that anxiety, depression, OCD symptoms, and insights of OCD patients worsened during the first COVID-19 lockdown, remained quite stable during a temporary elimination of the restrictions, and improved during the second pandemic-related lockdown. The changes observed in the whole sample were attributable to two specific subgroups of patients: those taking benzodiazepines and those with recent OCD onset.

In the entire study group, we observed a non-statistically significant worsening of obsessive–compulsive symptoms and a statistically significant worsening of insight during the first lockdown, compared to the pre-pandemic period. Subsequently, a significant improvement was observed in both OCD symptoms and insight between the temporary stop of the restrictions and the second lockdown. Slightly different patterns were observed for anxiety and depressive symptoms, which increased during the first lockdown but did not show significant decreases throughout the rest of the analyzed period.

There are different possible explanations for the described symptoms course. Clearly, the first lockdown was the event that impacted the psychopathological status of the patients the most, causing significant symptoms to worsen. This is consistent with other published studies on the short-term effect of the pandemic outburst [23]. We assume that the psychological distress produced by the sudden disruption of patients’ daily routines and the concomitant perception of a substantial threat to the survival of all mankind might account for this worsening. In fact, the increase of symptoms was observed in all the assessed psychopathological dimensions, even suggesting that it reflects an adjustment reaction not specific to the clinical population under study.

On the contrary, the fact that the severity of the symptoms remained unvaried during the temporary abolition of restrictions, and despite the optimistic claims concerning the epidemiological data, can be explained by the cognitive inflexibility and the hyper-prudential cognitive style characterizing OCD patients [24], that probably kept them away from the general looseness towards the risk of contagion that was present in Italy during the summer of 2020. In fact, we can hypothesize that OCD patients still perceived the risk of contagion as very high during that period. Consistently, the symptom improvement observed in concomitance with the imposition of the second lockdown can be interpreted as the effect of the sense of relief occurring when a rule patients considered highly necessary was promulgated. Moreover, it is possible that the succession of news represented an additional source of reassurance, spread during that same period (i.e., November–December 2020) that the vaccine against COVID-19 was almost ready to be marketed and that the beginning of the vaccination campaign was approaching.

Turning to the subgroup analyses that we have performed on our data, we observed that two clinical features of the patients in the examined sample were associated with the statistically significant changes of symptoms observed at the different time points: the benzodiazepines use at baseline (before the pandemic) and the duration of illness.

In fact, all clinical scores of patients taking benzodiazepines significantly increased during the first lockdown and significantly decreased during the second lockdown. In contrast, patients not using benzodiazepines did not show significant differences in any scale at any time point. Even if benzodiazepines are not a first-line treatment of OCD, their efficacy in this category of patients has been documented in scientific literature, as they were reported to reduce OCD symptoms and improve patients’ quality of life, suggesting that benzodiazepines can be an effective treatment for a subgroup of OCD patients with an anxious diathesis [25,26,27]. Consistently, a possible explanation for the findings of our study is that the benzodiazepine use might be a proxy of an anxious diathesis characterizing a subgroup of patients and that this proneness to anxiety made them more worried about the environmental circumstances and more susceptible to psychopathological worsening in case of adversities. In terms of clinical management and public health policies, this could imply that OCD patients taking benzodiazepines might deserve special attention in case of increased environmental stressors, being a population at higher risk of psychopathological worsening. It should be noted, however, that, in our sample, patients who used benzodiazepines were not balanced in number to patients who did not use benzodiazepines. The imbalance in favor of the former and the associated small sample size of the latter, may have somewhat biased the results, which should therefore be interpreted with caution.

Another clinical feature that greatly influenced the course of the symptoms during the pandemic was the duration of illness. In fact, only half of our sample, including the patients with a shorter duration of illness, showed a significant worsening of symptoms during the first lockdown and a significant improvement during the second one. On the contrary, the graphs showing the symptoms course of half of the sample with higher illness duration are virtually flat (Figure 3B). This finding might help elucidate some mechanistic aspects of OCD’s psychopathology and disease progression over time. In fact, our results suggest that at the beginning of the illness, the cognitive–emotional balance of OCD patients is more unstable and so susceptible to changes under the pressure of external circumstances. Afterwards, with the progression of illness, it is possible that the symptoms of OCD patients become less affected by the environment. This partial detachment from the potential stressors might have more than one explanation. Firstly, it is possible that the allostatic mechanisms that have developed since the onset of illness have led to a ceiling effect, which, in turn, makes further symptoms worsening less likely. Another possible explanation is that patients with chronic OCD become so entrenched in the intrinsic psychopathological mechanisms of their own OCD that they become less careful and less worried about external threats.

To our knowledge, there are no other published studies correlating the duration of illness with the response to environmental stressors in OCD patients. It is possible that the conflicting data among the published studies regarding the effect of the pandemic on OCD symptoms were due to differences in sample selection, particularly in terms of the duration of the illness.

Viewed as a whole, our results add information about the psychopathology of OCD by suggesting two specific factors that might influence the variability of symptoms of OCD patients under the pressure of adverse external circumstances, namely the anxious diathesis and the duration of illness. Besides the heuristic value of these observations, they also have possible practical implications, as they indicate two possible sub-populations of OCD patients more at risk of symptoms worsening in case of adverse contingencies and, therefore, deserving specific clinical attention. Moreover, should these results be confirmed in larger populations of OCD patients, the clinical features indicating a higher psychopathological risk could be included in more complex machine learning algorithms to be used for the purpose of public health policies [28].

Finally, from an evolutionary standpoint, it is possible to speculatively hypothesize that under specific circumstances, the anxious diathesis and the shorter illness duration could turn from being vulnerability factors to evolutionary advantages for those OCD patients displaying these features. In fact, a previous study from our group indicated that OCD patients who had previously displayed prevailingly checking symptoms during the first lockdown turned to hygiene-related ones and that new-onset pandemic-related OCD patients mainly displayed symptoms of hoarding essential goods and supplies. Since both hygiene-related and hoarding symptoms make the contagion factually less likely, we can hypothesize that the anxious diathesis and the shorter illness duration, by mediating the severity of symptom increase, could have been protective and evolutionarily advantageous if the COVID-19 had been even more deadly than it actually was, and the precautions taken by the general population had not been sufficient to protect them from the contagion.

The present investigation has certain methodological flaws. Primarily, the reduced sample size potentially prevented us from recognizing minor changes in symptom severity throughout the follow-up period. This numerical limitation arose from the short timeframe between the ethics committee approval and the end of the first mandatory lockdown in Italy. Indeed, due to the brief recruitment time and the unparalleled distress of the lockdown, a noteworthy proportion of patients identified through medical records were uncontactable or denied their participation, citing personal or family health-related grounds. This resulted in a diminished recruitment rate of approximately 45%. Therefore, the effect of a possible selection bias must be taken into account when interpreting the results of this study, as the sample included in our analysis might not be fully representative of the OCD population.

Moreover, the choice to exclude individuals who had not received psychiatric evaluations within the three months preceding the emergence of the pandemic limited the study population. The aim of this selection criterion was to reduce the risk of confoundings deriving from major life events that might have happened to the patients between a more distant time frame and the implementation of lockdown measures. However, despite the limited sample size, post hoc power analyses indicated the robustness of our findings. Another methodological shortcoming, possibly biasing the results of the study, is the adoption of different procedures for the collection of baseline data (clinical records) and follow-up ones (video calls). Moreover, we chose not to include in the study OCD patients with psychiatric comorbidities since our goal was to unveil specific mechanistic aspects of OCD psychopathology. However, considering the high rate of comorbidity characterizing the OCD population, the exclusion of patients with comorbid conditions might have hindered the generalizability of our results. Lastly, we did not perform a systematic evaluation of the type, dose, and duration of the benzodiazepines used in our sample, so our results regarding the influence of this drug treatment on the way to react to the pandemic of OCD patients should be considered preliminary and more in-depth and extensive studies are warranted to draw any conclusion.

## 5. Conclusions

The severity of psychopathological symptoms of OCD patients was affected by the COVID-19 pandemic. Fluctuations were observed according to the different phases of the pandemic and were most evident in OCD patients taking benzodiazepines and in those with recent-onset OCD. On one hand, these results point to specific subpopulations of OCD patients deserving special clinical attention in case of environmental adversities. On the other hand, they may suggest a hypothetical better chance of survival for these subpopulations in case of an extraordinary necessity of hygiene-related and hoarding behaviors, like during highly contagious and deadly pandemics.

## Figures and Tables

**Table 1:** Descriptive statistics of the overall group of OCD patients (*n* = 46) as a function of the data-collection time points.

	T0	T1	T2	T3
Sex (M/F)	24/22	/	/	/
Age (years)	37 (25)	/	/	/
Education (years)	13 (4)	/	/	/
Illness duration (years)	14.5 (19.25)	/	/	/
Y-BOCS	18.5 (24)	22 (16)	21 (16) ^b^	18 (40) ^b^
BABS	2.5 (9) ^a^	4.5 (11) ^a^	4.5 (10) ^b^	3 (9) ^b^
STAI-Y	47 (23) ^a^	56 (29 )^a^	50.5 (27)	49.5 (25)
BDI-II	10 (16) ^a^	14 (25) ^a^	15 (27)	13 (20)

Descriptive data are reported as median (IQR) for continuous variables and as counts for categorical variables. Univariate statistics are based on the Wilcoxon signed-rank test: ^a^ and ^b^ indicate significant differences between two time points. Abbreviations: BABS = Brown Assessment of Beliefs Scale; BDI-II = Beck Depression Inventory-II; F = female; IQR = inter-quartile range; M = male; OCD = obsessive–compulsive disorder; STAI-Y = State-Trait Anxiety Inventory-Y; Y-BOCS = Yale–Brown Obsessive Compulsive Scale.

## Data Availability

The original data presented in the study are openly available in Zenodo at: https://doi.org/10.5281/zenodo.10866651.
